# A latent class analyses of economic stability, medical mistrust, and barriers to care in relation to PrEP awareness in Black communities in Miami, FL

**DOI:** 10.1371/journal.pgph.0005379

**Published:** 2025-12-29

**Authors:** Devina J. Boga, Jordan Patrick, Kimberly Lazarus, Kayla Etienne, Nadine Gardner, George Gibson, Kalenthia Nunnally, Arnetta Phillips, Sannisha K. Dale

**Affiliations:** 1 Department of Psychology, University of Miami, Coral Gables, Florida, United States of America; 2 Flashlight of Hope, Miami, Florida, United States of America; 3 Blessing Hands Outreach, Miami, Florida, United States of America; University of Alabama at Birmingham, UNITED STATES OF AMERICA

## Abstract

In the U.S., Black communities are disproportionately impacted by HIV due to economic instability, racism, and structural inequities. Among Black residents who participated in a bundled implementation study from November 2019 through March 2020 in HIV high impact communities in Miami, FL this study examines (a) which latent classes are most prevalent based on indicators of economic stability (ES; income, work, housing, zip-code poverty thresholds), medical mistrust (MM), and barriers to care (BAC) and (b) the latent classes in relation to PrEP awareness and speaking with a doctor regarding HIV (e.g., testing, protection, treatment). Among 569 Black residents (mean = 42.4 years) 67% had never heard of PrEP, but 64% had spoken with a doctor about HIV. Four salient classes emerged: Class 1(34.8%) reported the second highest ES, lowest MM and least BAC. Class 2(18.7%) had the lowest ES, low MM and moderate BAC. Class 3(33.0%) exhibited the highest ES, high MM, and no BAC. Class 4(13.5%) had the third highest ES, highest MM, and highest probability for BAC. No significant differences between classes were found for PrEP awareness, but Class 1 had a higher likelihood of a provider conversation about HIV. Despite varying ES, MM levels and BAC, Black residents of HIV high impact communities are not being informed about PrEP. Interestingly, a significant percentage had provider conversations regarding HIV, especially Black residents with the highest ES, least BAC, and lowest MM. These findings also highlight the need for provider conversations with clients that discuss biomedical prevention tools such as PrEP.

## Introduction

The prevention, treatment and care related to HIV in Black communities in the U.S. is impacted by enduring barriers such as racism, housing stability, social and economic marginalization, and systemic inequities [1, 2]. In the United States (US), 38% of HIV incidence occurred among Black individuals in 2022 whilst only accounting for 13% of the US population [3]. This HIV-related health disparity persists throughout the prevention [including testing and use of Pre-Exposure Prophylaxis (PrEP)], treatment, and care continuum. Although oral PrEP was approved in 2012 in the US, there are substantially low rates of both awareness and utilization of PrEP in the Black community. In 2019, only 8.2% of Black individuals were engaged in PrEP use as compared to 63.3% of White persons and 14% of Latino/Hispanic individuals [4,5]. It is well established that health disparities experienced by the Black community are driven by social determinants of health, particularly in economic stability, health care access and quality, and social/community context [6,7]. However, it is less known how varying levels of these factors impact individuals, their PrEP awareness, and HIV knowledge.

In the domain of economic stability, studies have looked at a number of factors such as income, employment, education, neighborhood poverty levels and their relationship to HIV-related outcomes [7–9]. One study found that rates of HIV diagnoses were higher among individuals living in census tracts with higher poverty percentage, decreased income, and higher unemployment, and when further stratified by race these rates were disproportionately higher amongst Black communities [10]. Authors Wiewal et al [11] reported similar findings on the association between poverty and diagnosis rates and found it was stronger among women compared to men. When focusing on prevention efforts, such as speaking to a doctor regarding HIV and PrEP, a qualitative study revealed that Black women of middle socio-economic status felt that their providers rarely encouraged discussion of risk, prevention, or testing related to HIV requiring patients to self-advocate for these conversations [12]. Geographically, disparities in HIV prevention, diagnoses and care are concentrated in the Southern states [13]. In Florida, only 33.3% of health needs are met, deeming it a health professional shortage area [14,15]. Accessing PrEP has proven to be a complex and cumbersome task particularly for uninsured individuals, with frequent eligibility checks, numerous documents to be filled out, routine lab testing and the general lack of PrEP clinics where the necessity is greater [16,17]. Other barriers to care identified in the literature include societal level factors such as long distances to health facilities, anticipated costs of care, lack of transportation, and insufficient affordable housing [18,19].

When evaluating the current state of PrEP awareness, access, and HIV prevention related conversations with providers, it is imperative to consider the social and community context [20]. Data from several studies suggest that health seeking behaviors vary according to gender and health outcome [21–24]. Existing research recognizes the critical role played by medical mistrust in healthcare and institutions for those placed at risk and living with HIV [25–30]. Although medical mistrust is measured at an individual level, it is important to acknowledge that it is often a product of historical wrongdoings [31,32] and systemic oppression beyond the individual behavior and that it is maintained by the same system it was created from. A cross-sectional study looking at relationships between masculinity, medical mistrust and use of preventive services in Black/African men, found that men reporting higher medical mistrust were significantly more likely to delay screenings and health check-ups [33]. Ho, Sheldon, and Botelho (2022) conducted a scoping review on medical mistrust and health among marginalized women finding that 84% of the studies examined the intersection of gender and race, with the majority of studies reporting the negative impact of medical mistrust on health [34].

Although some studies have been carried out on economic stability, medical mistrust and barriers to care in relation to HIV prevention, many of them have been focused on sexually minoritized individuals and have not considered the nuanced variations in these indicators among groups [27,35–37]. The current study will use Latent Class Modeling to understand how many classes based on medical mistrust, barriers to care and economic stability exist among Black individuals and how they impact HIV prevention through PrEP awareness and speaking to a doctor regarding HIV. There are two hypotheses in this paper: (1) it is hypothesized that there will be statistically significant differences in classes (based on economic stability, mistrust, and barriers to care) between women and men therefore yielding different classes and (2) in classes presenting or experiencing higher economic stability and less barriers to care, there will be a positive association with HIV prevention (through PrEP awareness and speaking to doctor about HIV).

## Methods

### Ethics statement

This study was performed in accordance with the principles of the Declaration of Helsinki. All study procedures and materials were approved by the Institutional review Board at the University of Miami (9/16/2019, No. 20190791). Informed consent was obtained from all individual participants included in the study. Research participants have provided informed consent for the publication of the research findings in a peer-reviewed journal.

### Study design, study population, and procedures

Data in the current study come from piloting of the Five Point Initiative (FPI), a bundled implementation study that developed and harnessed the relationships between local community businesses/establishments, health partner organizations with mobile HIV testing and prevention capacity, community consultants working in tandem with the research team, and community residents who were living in specific zip codes that have the highest number of Black individuals living with or placed at risk for HIV. The FPI implementation strategy included innovatively collaborating with 5 categories of businesses: corner stores, laundromats, car service/mechanic shops, hair/beauty salons, and barbershops, to best reach Black residents in their own community. Cross-sectional surveying was conducted between November 1, 2019 and March 7, 2020. Eligible participants were those that were at least 18 years of age. Recruitment occurred through community-based advertisement and by engagement through community outreach specialists and study staff at the business location. If a potential participant expressed interest in completing the survey (available in English, Spanish or Haitian Creole), they were invited to complete the informed verbal consent with a study staff. For verbal consent, there was a script read out to each participant by the study staff. This script described what type of questions would be included, approximate time to complete the survey, the right to skip questions or end the survey if the participant wanted, and information on confidentiality, data sharing limits, and participation stipend. The participant had to expressively say yes or no to the study staff and the response was recorded. After being verbally consented all participants chose to continue, and no one declined.

At the end of the entire survey process the team member who assisted the participant also provided their signature and date on a receipt/verification document. Participants completed the self-report survey on their own or if asked/desired had the survey administered to them by a team member. All data was collected using a study iPad/tablet or the participant’s phone through a self-report survey and managed using REDCap electronic data capture tools hosted at the University of Miami [38,39]. After completion of the survey, the participant received a $15 to $25 voucher (depending on services/goods offered by the business) to spend at the partnering business. All eligible participants were offered educational materials, HIV testing, PrEP education, and condoms regardless of participation. The study was approved by the University of Miami Institutional Review Board.

## Measures

### Sociodemographic

The survey’s initial segment asked questions regarding age and gender identity (male/man, female/woman, trans male/man, trans female/woman, genderqueer/gender non-conforming/non-binary, or different identity). For analysis examining gender all woman (cis and trans) were grouped together and all men (cis and trans) were pooled together. Participants that identified as gender non-conforming/non-binary (n = 2) or different identity were set as missing.

### Economic stability

Three items describing an individual’s economic circumstances were included (*income, housing, and work status*) as well as one measure (*zip code level poverty thresholds*) as proxy for a community level indicator for economic circumstance.

*Income* At the individual level, one question asked about annual income with seven response options (<$5,000, $5,000 - $11,999, $12,000 - $15,999, $16,000 - $24,999, $25,000 - $34,999, $35,000 - $49,999, ≥ $50,000)

*Work status* Another item inquired about current work status [(full time work, part time work, full time or part time in school, neither in work nor in school, on disability)].

*Housing* One item asked about current housing arrangement with nine response options (renting home/apartment, living in home or apartment owned by you/someone else in household, residential drug, alcohol, or other treatment facility, publicly subsidized housing (like Section 8), a friend or relative’s home/apartment (pays little or no rent), temporary/transitional housing (e.g., hotel, HIV/AIDS specific housing, Sober Living), homeless: sleeping in a shelter, homeless: sleeping on the street, beach, car, etc., or other)], for analysis purposes, housing categories were further categorized into 5 categories that grouped similar categories (see Table 1).

**Table 1:** Demographic Characteristics of Latent Classes in Five Point Initiative Study.

Characteristic		Latent Classes				Test Statistic
Name (col%)	OVERALL	Class 1 n = 188(33.0)	Class 2 n = 198(34.7)	Class 3 n = 106(18.6)	Class 4 n = 77(13.5)	
**Age** (years; mean [standard deviation])	42.6(15.2)	43.3(15.2)	40.5(15.6)	42.7(14.1)	45.3(15.4)	F(3, 564) = 2.15
**Gender** ^**b**^						
Men	249(43.8)	88(47.4)	77(39.1)	43(40.9)	39(50.6)	χ^2^(3) =0.84
Women	320(56.2)	100(52.6)	120(60.9)	62(59)	38(49.3)	
**Education**^**c**^ **n(col%)**						χ^2^(21) =31.6
≤ 8^th^ grade	19(3.3)	11(5.8)	4(2.0)	2(1.9)	2(2.6)	
Some HS	92(16.2)	29(15.3)	26(13.2)	23(21.9)	14(18.2)	
HS graduate/GED	203(35.7)	57(30.0)	69(35.0)	48(45.7)	29(37.7)	
Some college	125(22.0)	37(19.5)	52(26.4)	16(15.2)	18(23.4)	
College graduate	74(13.0)	31(16.3)	28(14.2)	8(7.6)	9(11.7)	
Some graduate school	16(2.8)	9(4.7)	2(1.0)	2(1.9)	3(3.9))	
Graduate school degree	32(5.6)	13(6.8)	13(6.6)	4(3.8)	2(2.6)	
Choose not to answer	8(1.4)	3(1.6)	3(1.5)	2(1.9)	–	
**Relationship status** ^**b**^						
Married	44(17.7)	21(11.0)	13(6.6)	5(4.8)	5(16.7)	χ^2^(18) =21.6
Not married, living with partner	17(6.8)	5(2.6)	7(3.5)	4(3.8)	1(3.3)	
Non-cohabitating relationship	11(4.4)	3(1.6)	7(3.5)	1(0.9)	0(0.0)	
Single	9(3.6)	1(0.5)	3(1.5)	4(3.8)	1(1.3)	
Divorced/separated	147(59.0)	65(34.2)	40(30.3)	23(31.0)	19(24.7)	
Loss of long-term partner/widowed	10(4.0)	5(2.6)	3(1.5)	1(0.9)	1(1.3)	
Missing	11(4.4)	5(4.8)	3(3.9)	–	3(10.0)	
**Sexual Orientation** ^**b**^						
Heterosexual	477(83.8)	156(82.1)	167(84.8)	85(80.9)	69(89.6)	χ^2^(24) =19.2
Gay	8(1.4)	2(1.0)	2(1.0)	3(2.8)	1(1.3)	
Lesbian	11(1.9)	3(1.6)	3(1.5)	5(4.8)	0(0.0)	
Bisexual	23(4.0)	9(4.7)	7(3.5)	5(4.8)	2(2.6)	
Queer	5(0.9)	2(1.0)	2(1.0)	1(0.9)	0(0.0)	
Pansexual	2(0.3)	1(0.5)	0(0.0)	0(0.0)	1(1.3)	
Asexual	13(2.3)	4(2.1)	4(2.0)	2(1.9)	3(3.9)	
Unsure/Questioning/Exploring	13(2.3)	6(3.2)	6(3.0)	1(0.9)	0(0.0)	
Not listed	17(3.0)	7(3.7)	6(3.0)	3(2.9)	1(1.3)	
**HIV Status** ^**b**^						χ^2^(12) =11.5
HIV-positive, detectable viral load	9(4.5)	3(1.6)	3(1.5)	2(1.9)	1(1.3)	
HIV-positive, undetectable viral load	17(3.0)	4(2.1)	5(2.5)	3(2.9)	5(6.5)	
HIV-positive, I don’t know my viral load	6(1.0)	5(2.6)	1(0.5)	–	–	
HIV-negative	495(87.0)	162(85.3)	174(88.3)	93(88.6)	66(85.7)	
I don’t know	42(7.4)	16(8.4)	14(7.1)	7(6.7)	5(6.5)	

^a^Analysis of variance; ^b^χ^2^; ^c^Kruskal-Wallis test; *Exact p values < .05 considered statistically significant.

*Zip code level poverty thresholds* At the community level, zip code was utilized and matched to the US Census American Community Survey (ACS) [40] poverty status in the past 12 months variable assigning the percent living below poverty level value for each zip code. At the time of data cleaning the 5-year estimates were selected as the 1-year estimates were not available for the year 2020 and by each year of 2019 and 2020.

### Medical mistrust

The Medical Mistrust Index [41] (MMI) consists of 17 items of which 5 items used in this study gauge the general level of trust between a patient and the healthcare environment (Cronbach alpha for this sample = 0.77). An example item from this scale reads “Patients have sometimes been deceived or misled at hospitals”. A higher score indicated greater medical mistrust. The MMI has been used and validated in various health populations including HIV and among Black populations [42–44].

### Barriers to Care (BACS)

This scale 46 has 12 items that allows individuals to indicate barriers experienced in the context of geography, psychosocial factors, and resources in relation to healthcare services. The current study used 8 items (with 2 items adapted) from the scale with the aim of keeping brevity and generalizability for community administration. The participants were asked to respond by selecting yes or no for each barrier after this prompt “Please select which of the following have made it difficult for you to receive the healthcare services you need.” An example of a barrier was “long distances to medical facilities and personnel”. Previous research has demonstrated good reliability for BACS(α = 0.86-0.96) [45,46]. The Cronbach’s alpha for this sample was 0.80.

### HIV prevention

One item was asked to assess PrEP awareness with a binary response of yes or no: “Have you ever heard of PrEP (Pre-exposure Prophylaxis)?”. The other item assessed speaking with a doctor regarding HIV with a binary response of yes or no: “Have you ever spoken to a doctor about HIV (for example, regarding testing, protection against HIV, treatment for HIV, etc.)?”

### Data analytic strategy

In order to test our hypotheses, a corrected three-step approach Latent Class Analysis (LCA) technique was used [47]. In the first step, the unconditional LCA model was determined for the overall group and a multigroup statement was used to understand the moderating effect of gender on unconditional latent classes. In order to test if the men and women latent classes were statistically different, a scaling correction factor log-likelihood difference test was conducted. A model where classes were constrained to have identical thresholds across gender was used for the likelihood difference tests. Descriptive statistics were calculated for the gender sub-grouped and overall group using means and standard deviations for the continuous variables and frequencies and percentages for the categorical variables. Dependent on the results of the strictly positive Satorra-Bentler scaled chi-square difference test [48], it was determined that if no significant difference existed between groups that the next two steps of the approach continued only using the class group model that was constrained equal across gender, however if the models were significantly different, both multigroup and overall group would be reported. Both continuous and categorical indicators were used to conduct the latent class modeling. To determine best model fit, a variety of criterion were used including, Bayesian Information Criterion (BIC), Sample-size adjusted Bayesian Information Criterion (SSBIC), Lo-Mendell-Rubin likelihood ratio test (LMR-LRT) and entropy [49]. Models with lower BIC, SSBIC, higher entropy and a significant LMR-LRT p-value indicated better fit [49]. In the second and third steps, misclassification rates were calculated and the classes were analyzed with the covariates (HIV Prevention). To explore differences in sociodemographic characteristics across our classes, a series of Fisher’s exact test with Monte Carlo p-values were calculated for the categorical variables and for age (continuous) we used a one-way ANOVA procedure. To rank each class according to their overall economic stability, the probabilities of each categorical indicator were summed according to two groups of unstable versus stable using various references to determine stability (i.e., living wage calculator) [9,50–52]. All data management and analyses were conducted using SAS software v9.4 for Windows (SAS Institute, Cary, NC, USA), and Mplusv8.6 [53,54]. For analyses, we included only individuals who identified as Black and/or African American, which were the vast majority of the total sample (n = 677).

## Results

### Sample characteristics

#### Overall sample.

The final overall sample used in analysis included 569 Black participants with a mean age of 42.6 years and a standard deviation of 15.2 years. As shown in the first column in Table 1, over half of the sample identified as women (56.2%), followed by men (43.8%). As previously mentioned for analyses the genders were grouped into men (cis and trans [n = 2]) and women (cis and trans [n = 1]) and genderqueer/gender non-conforming/non-binary (n = 2) were coded as missing. The analytic sample identified as Black (100.0%), with almost 80% of participants being born in the US. English was the most spoken language (97.9%), followed by Kreyol (9.7%), and Spanish or other (5.8%). Most of our sample described their sexual orientation as heterosexual (83.3%), almost sixty percent of the sample was divorced or separated and 35.7% were a high school graduate or obtained a GED. In testing sociodemographic characteristics (non-indicator variables in the LCA) differences amongst classes, only relationship status was significantly different between classes as shown in Table 1. In our overall sample, we found that 67% of the participants had never heard of PrEP, but 64% had spoken with a doctor about HIV.

### Latent class analysis results

**Fig 1:**
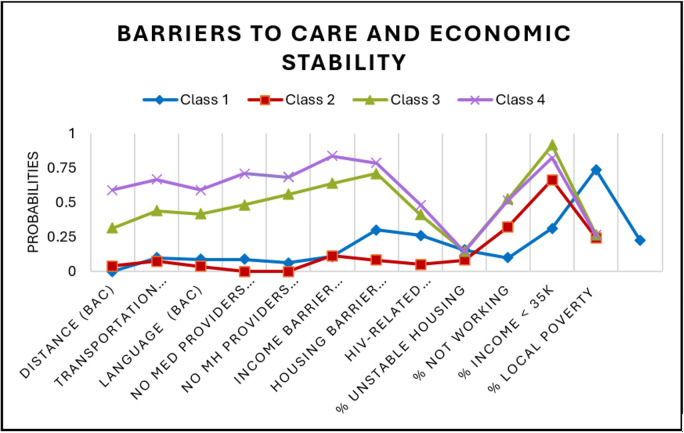
Unconditional Model Probabilities for Barriers to Care and Economic Stability for Overall Sample. Shown are distributions across four classes. Barriers to care listed include distance, transportation, language, lack of medical providers, lack of mental health providers, income, and HIV-related stigma. Economic stability is represented by percents for unstable housing, not working, with income less than $25,000, and local poverty.

**Fig 2:**
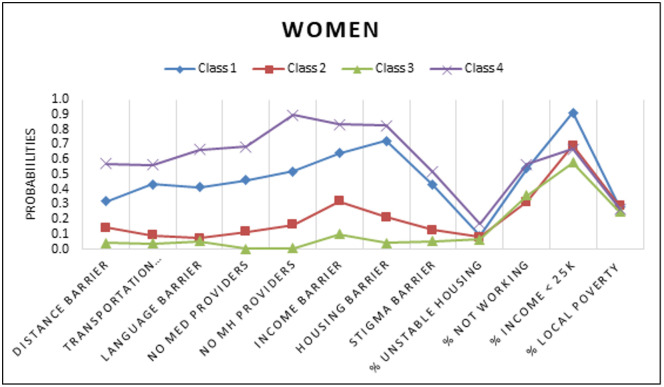
Unconditional Model Probabilities for Barriers to Care and Economic Stability among Women. Shown are distributions across four classes. Barriers to care listed include distance, transportation, language, lack of medical providers, lack of mental health providers, income, and HIV-related stigma. Economic stability is represented by percents for unstable housing, not working, with income less than $25,000, and local poverty.

**Fig 3:**
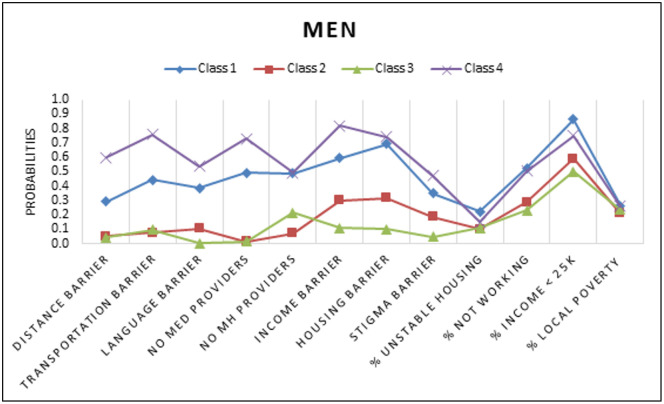
Unconditional Model Probabilities for Barriers to Care and Economic Stability among Men. Shown are distributions across four classes. Barriers to care listed include distance, transportation, language, lack of medical providers, lack of mental health providers, income, and HIV-related stigma. Economic stability is represented by percents for unstable housing, not working, with income less than $25,000, and local poverty.

**Fig 4:**
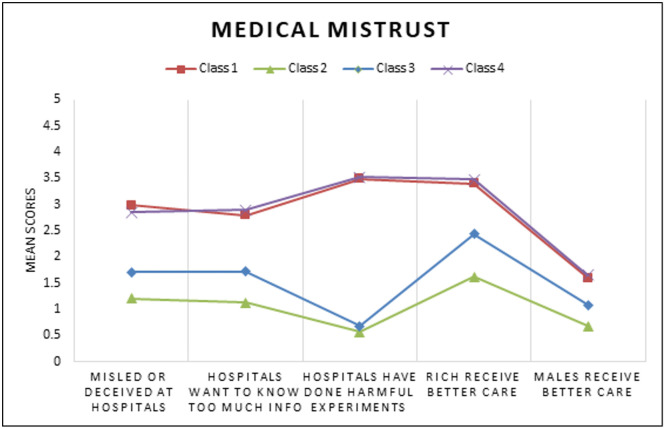
Unconditional LCA Model for Medical Mistrust Means among Overall Sample.

**Fig 5:**
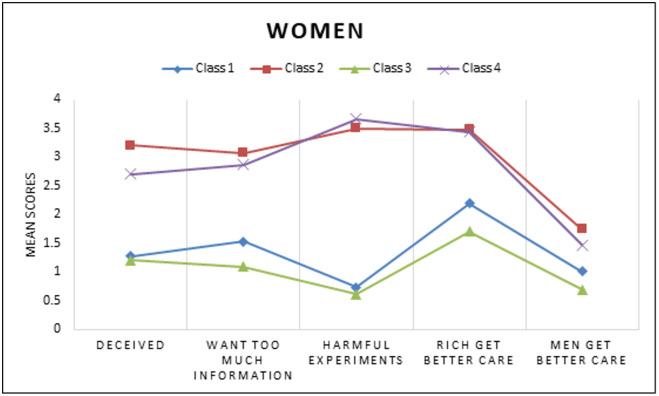
Unconditional LCA Model for Medical Mistrust Means among Women.

**Fig 6:**
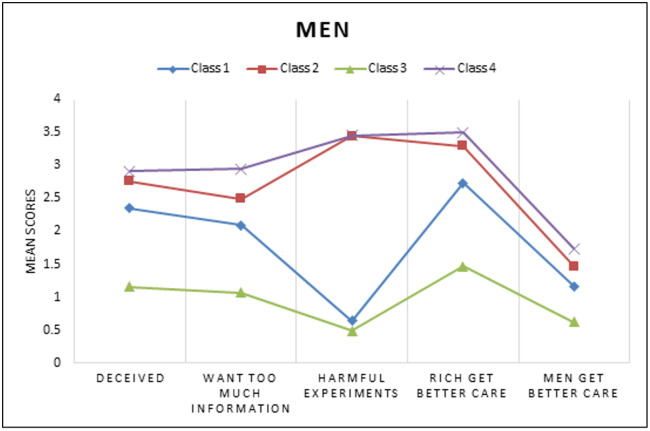
Unconditional LCA Model for Medical Mistrust Means among Men.

A series of models for overall and gender subgroups were estimated. Table 2 depicts the standardized solution of the unconditional models that were repeated and compared with increasing numbers of unconditional classes using BIC, SSABIC, LMR-LRT, and entropy fit criteria for the full sample (model 1). A 4-class model solution was chosen as indicated based low BIC and SSABIC, high entropy and significant p-value LMR-LRT suggesting that the 4-class model significantly improved the fit compared to the 3 class model (p < .05) [49]. For the final overall group model, the posterior probabilities were 34.8% for class1, 18.6% for class 2, 33.0% for class 3, and 13.5% for class 4. The next model (model 2) estimated was the conditional model based on gender (without the HIV Prevention outcomes of interest), in this model class proportions were restricted to be the same across classes for gender. In this test it was determined that there were significant differences between women and men groups across the latent indicators (χ^2^ = 142.68, p < 0.05). The third model (model 3) was estimated with 4 classes and gender with the outcomes of interest. A four-class solution was retained for the last model, adding a known class statement of gender produced different but similar classes as our second constrained model. Class probabilities and means of the unconditional model were depicted through six figures. Figs 1–3 depict the barriers to care and economic stability probabilities while Figs 4–6 depict medical mistrust scores among overall, women, and men, respectively. Each overall class is described below, but descriptions on the subgroup of women and men per class is provided in Table 3.

**Table 2:** Fit Statistics for Latent class models overall group (n = 569).

# of profiles	BIC	SSABIC	Entropy	LMR-LRT (p-value)	Class percentages
1	24109.138	24001.203	–	–	
2	23354.195	23154.199	0.806	938.9 (0.121)	45.1, 54.8
3	23022.345	22730.287	0.844	515.8(0.074)	25.8, 40.9, 33.2
4	22861.889	22477.769	0.909	344.4(0.003)	34.8, 18.6, 33.0, 13.5
5	22831.859	22355.678	0.894	214.0(0.241)	24.1, 18.3, 13.2, 18.4, 26.0

*BIC* Bayesian Information Criteria, *SSABIC* Sample size adjusted BIC, *LMR-LRT* Lo-Mendell-Rubin likelihood ratio test.

**Table 3:** Descriptions on subgroups of women and men in each latent class.

	Women	Men
Latent Class 1		
	*Low medical mistrust, moderate barriers to care, lowest economic stability*	*Low medical mistrust, moderate-high barriers to care, lowest economic stability*
	*n = 66, 20.8% of subgroup of women*	*n = 46, 18.5% of subgroup of men*
	Across all classes and both genders, women in this class were most likely to have an income less than $5,000 and were more likely to experience barriers related to shortages of mental health professionals, personal financial resources, and lack of adequate or affordable housing. Notably, this class had the highest percent living below poverty level (27.9%) among the women classes, as well as higher than the highest percentage living below poverty in the subgroup of men.	Class 1 had the highest percent living below poverty within group (26.3%). Medical mistrust averages were low (ranging between 0.64 - 2.74), with the highest probability of mistrust relating to better care for ‘rich’ patients compared to ‘poor’ patients. The barriers to care most likely to be endorsed in this class were related to personal financial resources and lack of adequate and affordable housing. Similarly, to the subgroup of women, this class exhibited the lowest economic stability. Across all four classes, they had the highest proportion of participants to be most likely earning an income of less than $5,000, most likely to be on disability or unemployed, highest probability to live in unstable housing and live in the neighborhood with the highest percent living below poverty level.
Latent Class 2		
	*Highest medical mistrust, low barriers to care, high economic stability*	*High medical mistrust, low-moderate barriers to care, high economic stability*
	*n = 98, 30.8%*	*n = 86, 34.5%*
	The women in class 2 were most likely to average higher scores on the medical mistrust items (1.75- 3.48) and less likely to endorse yes to the barriers of care. The most likely barrier of care was related to personal financial resources. Class 2 was most likely to live in the lowest percent living below poverty level zip codes, had a high probability of living in stable housing such as renting or a home that is owned by someone in the household, and were also more likely to have full time or part-time jobs and higher incomes.	The class 2 subgroup of men had the second highest average scores across the five medical mistrust items. The highest average score within the class was found for the “Hospitals have sometimes done harmful experiments on patients without their knowledge” item (mean = 3.44). The barriers to care most likely to be experienced by this class include barriers related to affordable and adequate housing and personal financial resources. This class had the lowest percent living below poverty level with slightly more than half of the participants more likely to be earning less than $25,000 annually. Additionally, a higher probability of individuals were more likely to be full time or part time workers and living in a home that was owned by themselves or a family member.
Latent Class 3		
	*Lowest medical mistrust, lowest barriers to care, highest economic stability*	*Lowest medical mistrust, lowest barriers to care, highest economic stability*
	*n = 117, 36.8%*	*n = 72, 28.9%*
	The women in class 3 had the lowest averages of medical mistrust across all items within and across the classes with averages ranging from 0.61 to 1.69, the highest average belonged to the item of rich patients receiving better care than poor patients. This class was least likely to endorse any barriers of care both within and across classes. The women were labeled as highest economic stability as they were more likely to live in areas with lower percent living below the poverty threshold (24.4%), lived in the most stable housing, and were most likely to have full time jobs and incomes that were greater than $5,000, with the highest probability across classes to have incomes in the $50,000 or greater category.	Similarly, to class 3 in the women group, this class exhibited the lowest medical mistrust, the average mistrust scores for all items were lower within the class, across classes and across the gender subgroups (means ranging from 0.48-1.46). The lowest probabilities for barriers to care were found in this class barring the lack of mental health providers barrier to care which was higher compared to class 2 for the men. For economic stability, this class was most likely to live in lower poverty threshold areas (23.5%), had the highest probability for renting an apartment or home, and the highest probability for working full time across classes within the subgroup of men. The highest proportion of probabilities of having an income of $35,000 or greater was found in this class.
Latent Class 4		
	*High medical mistrust, highest barriers to care, low economic stability*	*Highest medical mistrust, highest barriers to care, low economic stability*
	*n = 37, 11.6%*	*n = 45, 18.1%*
	We found high average scores of medical mistrust in class 4, the highest average mistrust score (m = 3.66) was attributed to the item stating that “hospitals have sometimes done harmful experiments on patients without their knowledge.” Compared to all other subgroup classes of women and across both genders, this class had the highest probability of experiencing all the barriers to care. This class had the second highest percentage of living below poverty (26.9%), higher probabilities of living in unstable housing (e.g., temporary/residential drug/unhoused), higher probabilities of being neither in work nor school, and higher probabilities of having an income greater than $25,000.	Amongst all subgroup classes of men the highest averages of medical mistrust were found in class 4 (averages ranging 1.74 to 3.50). Participants in this class had a higher probability of experiencing all of the barriers to care. This class was labelled as low economic stability as they were more likely to live in unstable housing (residential/temporary living), more likely to have an income less than $25,000, and less likely to have a full time or part time job.

#### Class 1.

***Overall: High medical mistrust, no barriers to care, and highest economic stability.*** The first class consisted of 33.0% of the sample (n = 188). As shown in Fig 4, class 1 exhibited the second highest average medical mistrust, with the highest average mean for the item “Patients have been misled or deceived at hospitals” compared to all other classes. This class also had a higher probability of endorsing “not experiencing” any barriers to care (Fig 1). For economic stability, class 1 is characterized as highest economic stability due to living in the least percent living below poverty level (M = 22.7, SE = 0.7), having a high probability of either renting an apartment or home, and being most likely to either have full-time or part-time jobs.

#### Class 2.

***Overall group: Lowest medical mistrust, no barriers to care, and high economic stability.*** The second class had 34.7% of the sample (n = 198), the participants in this class reported the lowest medical mistrust averages across all 5 items and they were more likely to endorse “not experiencing” in response to all the barriers to care items. In the economic stability indicators (percent living below poverty, housing, work status and income), this group was in the 2nd lowest group of percent living below poverty (M = 24.6, SE = 0.74) (meaning higher economic stability on a neighborhood level), the individuals had the highest likelihood to be renting an apartment or home compared to the other classes followed by living in a home or apartment owned by themselves or someone else in the household or staying at a relatives or friend’s home (paying little to no rent). The individuals in this group were categorized by having the highest probability of working part-time, and higher probabilities of being neither in work nor school or living on disability. This class had a higher probability (similar in magnitude) of either having an income ranging between $0 and $5,000 or $50,000 and greater. In summary this class can be described as the lowest mistrust, second highest economic stability, and no barriers to care.

#### Class 3.

***Overall group: Low medical mistrust, some barriers to care, and lowest economic stability.*** The pattern of lower medical mistrust, and probabilities of experiencing barriers to care that were specifically related to a lack of healthcare professionals that are adequately trained or knowledgeable, shortages of mental health providers, personal financial resources, and lack of adequate and affordable housing were found in Class 3 (n = 106, 18.6%). As shown in Fig 1, this class included individuals living in the highest level of percent living below poverty level compared to the other classes (Mean = 26.7, Standard Error = 1.0). For housing, they had a higher probability of renting a home or apartment, followed by living in a home or apartment owned by themselves or someone (paying little to no rent). Class 3 had the highest probability of working full time compared to the other classes. Individuals in this class were most likely to have an income of less than $5,000 within and across groups. This group was characterized as comparatively low economic stability due to the highest probability of working full time with lowest income, living in the highest poverty threshold and most likely to be renting an apartment or home.

#### Class 4.

***Overall group: Highest medical mistrust, most barriers to care, and lower economic stability.*** Class 4 comprised of 13.5% of the sample (n = 77). Individuals in class 4 endorsed the highest medical mistrust of all the classes, they also had the highest probability of experiencing all the barriers to care barring the last item regarding community stigma against people living with HIV (Figs 1 and 4). In terms of economic stability, the average percent living below the poverty level in this group was 26.2 (SE = 1.0), just slightly lower than class 2. In the individual level indicators of housing, work and income, this group had the highest probability of renting or owning a home or apartment, they also had a comparatively higher probability of living in a residential drug, alcohol/other treatment facility, or temporary/transitional housing. This class was more likely to be neither in work nor school or living on disability and had a higher probability of having an income between $0 and $15,999.

### Linking class membership and HIV prevention

#### Overall group.

Two different variables (PrEP awareness and speaking to a doctor about HIV) were added into the model to test the equality of probabilities across the 4 latent classes. PrEP awareness [overall model: χ^2^(3) = 1.95, p = 0.58] was not significantly different across any of the classes. For the probability of having spoken to a doctor regarding HIV (testing, prevention, treatment etc.) [overall model: χ^2^(3) = 13.44, p < .05)], class 2 (which was described as lowest medical mistrust, no barriers to care and second highest economic stability) was the group that was significantly more likely to speak to a doctor regarding HIV compared to class 3 (low medical mistrust, second most barriers to care, lowest economic stability) [χ^2^ (1) = 8.42, p < 0.05)] and class 4 (highest medical mistrust, most barriers to care, lower economic stability) [χ^2^ (1) = 9.45, p < 0.05].

#### Multigroup.

With the methods limitation of 1 latent variable in the model, the sample was subgrouped and two different models were estimated taking into account the start values of the first model where gender was allowed to vary on class and the outcome variables were added into this model. No statistically significant differences were found in the overall model across the four classes [χ^2^ (3) =0.68, p = 0.88] for PrEP awareness in the subgroup of women. Although the overall model for spoken to doctor was non-significant [χ^2^ (3) = 5.87, p = 0.12], the difference between class 2 and 3 showed significance [χ^2^ (1) = 5.32, p < 0.05] for women. PrEP awareness was not significantly different amongst the classes in the subgroup of men [overall test: χ^2^ (3) = 1.26, p = 0.74]. Significant differences were found between classes for speaking to a doctor regarding HIV related prevention and treatment [overall test: χ^2^ (3) = 7.85, p < 0.05] in the subgroup of men. The individuals in class 3 who exhibited high medical mistrust, low-moderate barriers to care, and high economic stability were significantly more likely to speak to a doctor compared to individuals in class 4 who were labeled with the highest medical mistrust, highest barriers to care, low economic stability [χ^2^ (1) = 5.67, p < 0.05]. Those in class 3 who were described as lowest medical mistrust, lowest barriers to care, highest economic stability were also more likely to speak to a doctor regarding HIV prevention compared to individuals in class 4 [χ^2^ (1) = 4.95, p < 0.05].

## Discussion

The novel findings of the overall group analysis showed that despite variations in economic stability, medical mistrust, and barriers to care we found a lack of association with PrEP awareness. We hypothesized that the group of women and men would be different in LCA modeling, however the results of lack of association with PrEP awareness was echoed in the multigroup analysis. Recent studies looking at PrEP awareness among Black cisgender men largely focus on gay, bisexual or men who have sex showing higher PrEP awareness possibly due to targeted education campaigns [37,55] Our study represented approximately 88% heterosexual cis-gender men and 80% heterosexual cisgender women, both groups of whom were not represented (by race and/or gender) in earlier efficacy trials or campaign efforts [56,57] One previous study found that in 218 participants, of those who identified heterosexual 81% were unaware of PrEP [58]. Although the overall finding is aligned with the current statistics of the insufficient PrEP coverage (percentage representing the number of individuals prescribed PrEP divided by those that have indications of PrEP) which amongst Black individuals, between January 2019 and March 2021 were only 7.9% and 9.0%, respectively, it also gleans insight into our knowledge surrounding the relationship between gaps in better healthcare and amongst groups living with lower and higher economic stability.

We hypothesized that the classes exhibiting higher economic stability and less barriers to care would have associations with HIV prevention. This finding was only true for prevention in the method of speaking to a doctor regarding HIV. In the overall group analysis, findings revealed groups with less barriers to care and higher economic stability were more likely to speak to their doctors compared to groups with more barriers to care and low/lowest economic stability. However, this relationship did not extend to PrEP awareness which beckons understanding of the nature of conversations with health providers surrounding HIV and the lack of focus on PrEP as a prevention method. Notably the multigroup analysis subgroup of men revealed that variations in medical mistrust did not significantly influence the likelihood of speaking to a doctor about PrEP. Findings in this study encourage intervention with providers. Interventions aimed at improving providers’ ability to engage in PrEP conversations are needed as well as on-going training to keep providers up to date on PrEP eligibility, usage, and modalities. In addition, strategies and interventions are needed to address barriers to care, provide PrEP knowledge and linkage, and meet community members in the neighborhoods where they live and socialize.

Four types of economic indicators were used to capture a comprehensive measure of economic stability. It would be remiss to not address the nuance and restriction within the measurement of housing. While renting is classified as stable, it does not account for challenges, such as payment arrears, risk of eviction, or high rent that hinders meeting other needs [59]. Structural racism through discriminatory systems such as redlining and gentrification has limited homeownership in Black communities. Renting represents a level of stability, especially in South Florida with rocketing rent prices. According to the living wage calculator created by the Massachusetts Institute of Technology [52], in Miami-Dade County an income of at least $39,353 is considered a living wage assuming that an individual is working full time, across all classes in overall and multigroup models at least half of the participants were making less than $25,000 annually.

Due to methodological limitations of two latent categorical variables not permitted to be present within the same model, the auxiliary three-step procedure was not used for the gender subgroup model. Therefore, HIV prevention outcomes of PrEP awareness and speaking to a doctor regarding HIV were regressed on different models where it was women only and men only. Start values were provided to guide the model to be as close to the class varying by gender initially estimated however the estimates produced were not an exact match, so the output must be interpreted with caution for the gender sub-grouped and outcome model. Also, we are using cross-sectional data and therefore can only identify associations not infer causality. Lastly, due to the low frequency of gender diverse (e.g., transgender, nonbinary) individuals in our sample all women (transgender and cisgender) and all men were grouped together. Future research with a larger frequency of gender diverse individuals should be conducted to better understand the role of gender in the latent classes and outcomes explored in this paper.

Our findings add to the HIV prevention literature by stressing the importance of structural barriers particularly economic instability on HIV prevention outcomes. Latent classes were associated with speaking with a doctor regarding HIV and differences between classes were found to be most strongly explained by economic instability. Medical mistrust, barriers to care, and economic stability were not associated with PrEP awareness in our study, indicating the general lack of awareness of PrEP in the community. There is a clear need for intervention at the provider level to improve PrEP education and increase advocacy and support in healthcare relationships regarding HIV prevention in the Black community.
